# Hypoxia-Inducible Factor-1*α* Regulates High Phosphate-Induced Vascular Calcification via Type III Sodium-Dependent Phosphate Cotransporter 1

**DOI:** 10.1155/2024/6346115

**Published:** 2024-03-26

**Authors:** Chengkun Guo, Zhengli Quan, Jingjing Ke, Hualong Zang, Qiuping Teng, Xin Li, Dan Peng, Ping Wang

**Affiliations:** ^1^Nephrology Department, Jingmen Central Hospital Affiliated to Hubei Minzu University, Jingmen, Hubei 448000, China; ^2^Neonatology Department, Jingmen Central Hospital Affiliated to Hubei Minzu University, Jingmen, Hubei 448000, China

## Abstract

Vascular calcification (VC) has a high incidence in patients with chronic kidney disease, which is a worldwide public health problem and presents a heavy burden to society. Hypoxia-inducible factor (HIF)-1*α*, the active subunit of HIF-1, has been reported to play a vital role in high phosphate-induced VC. However, the underlying mechanism is still undetermined, and effective treatment is unavailable. In the present study, human aortic smooth muscle cells (HASMCs) were cultured under normal or high phosphate media conditions. HIF-1*α* small interfering RNA and overexpression plasmids were employed to regulate HIF-1*α* expression. Phosphonoformic acid was employed to restrain the function of type III sodium-dependent phosphate cotransporter 1 (Pit-1). The expression levels of HIF-1*α*, Pit-1, runt-related transcription factor 2 (Runx2), and smooth muscle 22 alpha (SM22*α*) were evaluated, and the calcium contents were also examined. Cell growth was assessed using an MTT assay. High phosphate stimulation caused an upregulation in HIF-1*α* and Pit-1 expression levels and induced calcium depositions in HASMCs. Upregulation of Runx2 expression accompanied by downregulation of SM22*α* expression was observed in the high phosphate group. Following the suppression of HIF-1*α* expression, there was a concomitant attenuation in Pit-1 expression, calcium deposition, the alteration of phenotypic transition marker genes, and *vice versa*. The most serious calcium deposition was noted in HASMCs cultured under high phosphate conditions which were pretreated with a HIF-1*α* overexpression plasmid. However, when the biological functions of Pit-1 were restrained, the putative serious calcium deposition was not formed even in HASMCs transfected with a HIF-1*α* overexpression plasmid. The findings confirmed that HIF-1*α* regulated Pit-1 expression and exerted its pro-calcifying effect through Pit-1, which identified HIF-1*α* and Pit-1 as therapeutic targets for high phosphate-induced VC.

## 1. Introduction

Vascular calcification (VC) has a high incidence in patients with chronic kidney disease (CKD), which is a worldwide public health problem and presents a heavy burden to society [1–3]. Hyperphosphatemia is an essential triggering factor of VC; however, the underlying mechanism is still uncertain, and effective treatment is absent [4, 5]. The investigation of the molecular mechanism in VC that can be used to develop therapies has become a major focus of attention [6].

It has been previously shown that VC gradually progresses passively with the deposition of minerals. However, it has been widely accepted that VC is an active cell-mediated process, which includes the vascular smooth muscle cell (VSMC) phenotypic transition, calcification prompting factors and inhibitor disorders, apoptosis, and the dysfunction of the extracellular matrix [7–9]. In human VSMCs, type III sodium-dependent phosphate cotransporter 1 (Pit-1) is the major sodium-dependent inorganic phosphorus (NaPi) cotransporter, which plays a key role in high phosphate-induced VC [10, 11]. When Pit-1 is activated, intracellular inorganic phosphorus transport in VSMCs will be increased, and the downstream signal of Pit-1, such as RUNX2, will be activated, eventually leading to the occurrence of VC [10–13]. However, little is known regarding the regulation of Pit-1.

Hypoxia-inducible factor 1 (HIF-1) is a pivotal protein produced under hypoxic conditions, which participates in various biological processes, such as hematopoiesis, angiogenesis, inflammation, and tumor formation [14]. The activity of HIF-1 is mainly determined by its alpha subunit (HIF-1*α*) [15]. Although HIF-1*α* has been reported to perform a vital catalytic role in high phosphate-induced VC, the precise mechanism of this process remains unknown [16]. Based on existing literature and our previous research achievements, we hypothesized that HIF-1*α* may play a role in the regulation of VC through Pit1, which has not been reported yet.

The present study aimed to explore the interaction between HIF-1*α* and Pit-1 in high phosphate-induced VC and to examine the mechanisms involved in this process.

## 2. Materials and Methods

### 2.1. Cell Culture and Calcification Model

The human aortic smooth muscle cells (HASMCs) were purchased from Procell Life Science & Technology Co., Ltd. (Wuhan, China, cat. No. CL-0517), and their culture conditions were the same as described previously [12]. Na_2_HPO_4_·12H_2_O and NaH_2_PO_4_·2H_2_O were employed to simulate high phosphate conditions (2.5 mM; pH 7.2–7.4) in Dulbecco's modified Eagle's medium (HyClone; Cytiva) and induce VC as described previously [12]. HASMCs were divided into five groups as follows: (1) control (CNT) group, which contained HASMCs treated with normal inorganic phosphorus concentration (Pi; 0.9 mM); (2) high Pi group (HP), including HASMCs treated with high Pi (2.5 mM); (3) small interfering RNA (siRNA) and high Pi group (HPSI), which contained HASMCs transfected with HIF-1*α* siRNA that were treated with high Pi; (4) overexpression and high Pi group (HPOE), which included HASMCs transfected with HIF-1*α* overexpression plasmid that were treated with high Pi; (5) overexpression high Pi and phosphonoformic acid (PFA) group (HPOEPFA), which contained HASMCs transfected with HIF-1*α* overexpression plasmid that were treated with high Pi and 0.5 mM PFA. The media were renewed every other day, and the cells were cultured for a maximum period of 7 days.

### 2.2. Quantification of Calcification

The Ca^2+^ concentration of the cells was examined by a commercially available kit (Calcium assay kit; Nanjing Jiancheng Bioengineering Institute) as described previously [12].

### 2.3. Alizarin Red Staining

HASMCs were analyzed using standard Alizarin red staining as described previously [12]. Red or brown staining, as viewed under a light microscope (Olympus Corporation; magnification ×200), indicated positive staining of calcium nodules.

### 2.4. Reverse Transcription-Quantitative PCR (RT-qPCR)

TRIzol® reagent (Invitrogen; Thermo Fisher Scientific, Inc.) was used to isolate the total RNA from the tissue. The temperature protocol of the reverse transcription, the quantitative PCR conditions, and the primers were the same as described previously [12, 17]. The expression levels of the genes were detected with the 2^−ΔΔCq^ method [18]. The PCR primers were designed as follows: HIF-1*α*, 5′-CCGATGGAAGCACTAGACAAAGT-3′ (forward), 5′-TTTGAGGACTTGCGCTTTCAG-3′ (reverse); Pit-1, 5′-ACATCCTACACCATGGCAATAT-3′ (forward), 5′-CACTTCAGGCTTATCCTGATCAT-3′ (reverse); Runx2, 5′-TACTCTGCCGAGCTACGAAATG-3′ (forward), 5′-TGAAACTCTTGCCTCGTCCG-3′ (reverse); SM22*ɑ*, 5′-ATCCAAGCCAGTGAAGGTGC-3′ (forward), 5′-ACTCCCTCTTATGCTCCTGGG-3′ (reverse); GAPDH, 5′-CGCTAACATCAAATGGGGTG-3′ (forward), 5′-TTGCTGACAATCTTGAGGGAG-3′ (reverse).

### 2.5. Western Blotting

The western blotting procedure and conditions and the primary and secondary antibodies were the same as described previously [12, 17].

### 2.6. Cell Transfection

siRNA was employed to knock down the expression levels of HIF-1*α*, the sense sequence used was 5′-CUAUGACCUGCUUGGUGCUGAUTT-3′, and the antisense sequence used was 5′-AUCAGCACCAAGCAGGUCAUAGTT-3′. The transfections were performed using HiPerFect transfection reagent (Qiagen AB) following the manufacturer's instructions. Both normal cells and cells transfected with scramble siRNA were used as controls.

The overexpression plasmid of HIF-1*α*, pcDNA3.0-HA-HIF1A(human)-1, was purchased from the MiaoLing Plasmid Sharing Platform (P23864; MiaoLingbio), and the cells were transfected with X-tremeGene Transfection Reagent (Roche Diagnostics) according to the manufacturer's instructions. Both normal cells and cells transfected with empty vector plasmid were used as controls.

### 2.7. MTT Assay

The cells in each group were seeded at a density of 6000 cells per well in 96-well plates and cultured under either normal or high phosphate conditions. The culture media were refreshed every other day, and the cells were maintained for a maximum period of 7 days. Prior to testing, 10 *μ*l of MTT (3-(4,5-dimethyl-2-thiazolyl)-2,5-diphenyl-2H-tetrazolium bromide) was added to each well followed by an additional incubation period of 4 h. Subsequently, the culture medium was discarded, and the cells were treated with 150 *μ*l DMSO. The colorimetric analysis of samples was conducted using an enzymatic reader at a wavelength of 490 nm. All experiments were performed in triplicate.

### 2.8. Statistical Analysis

Every experiment was performed at least in triplicate. The data are shown as mean ± standard deviation. Statistical analyses were performed using SPSS 18.0 software (SPSS, Inc.). The differences between groups were evaluated using one-way ANOVA followed by Tukey's post-hoc test. *p* < 0.05 was considered to indicate a statistically significant difference.

## 3. Results

### 3.1. Effects of siRNA and Overexpression Plasmid on HIF-1*α* Expression

**Figure 1:**
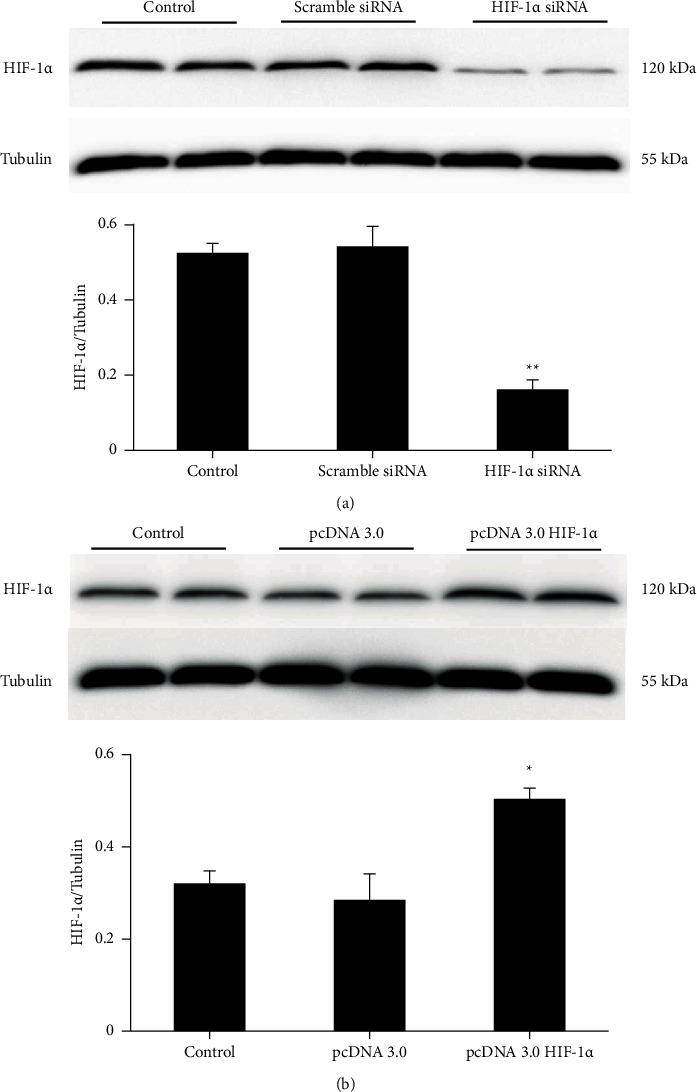
Changes in HIF-1*α* protein expression. HASMCs were cultured in normal DMEM media and transfected with HIF-1*α* siRNA (a) or an overexpression plasmid (b). The expression levels of the HIF-1*α* protein were examined by western blot analysis at 24 h following transfection. The levels were normalized to those of tubulin. The data shown are indicative of mean ± SD. ^∗^*p* < 0.05 vs. control; ^*∗∗*^*p* < 0.01 vs. control. HIF-1*α*, hypoxia-inducible factor 1 alpha; HASMCs, human aortic smooth muscle cells; DMEM, Dulbecco's modified Eagle's medium; siRNA, small interfering RNA.

**Figure 2:**
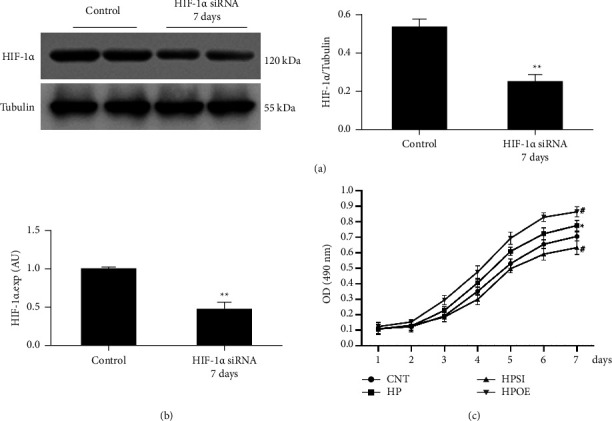
Knockdown efficacy and cell growth curve. The expression levels of HIF-1*α* protein (a) and mRNA (b) were examined by western blot analysis and RT-qPCR on day 7 following HIF-1*α* siRNA transfection. An MTT assay was used to assess the cell growth curve under different conditions (c). The data shown are indicative of mean ± SD. ^**#**^*p* < 0.05 vs. CNT; ^*∗*^*p* < 0.05 vs. HPSI; ^*∗∗*^*p* < 0.01 vs. control. CNT, HASMCs cultured under normal conditions with 0.9 mM Pi; HP, HASMCs cultured with 2.5 mM Pi; HPSI, HIF-1*α* siRNA-transfected HASMCs cultured with 2.5 mM Pi; HPOE, HIF-1*α* overexpression plasmid-transfected HASMCs cultured with 2.5 mM Pi. HIF-1*α*, hypoxia-inducible factor 1 alpha; siRNA, small interfering RNA; RT-qPCR, reverse transcription-quantitative PCR; SD, standard deviation; CNT, control; HP, high Pi; HASMCs, human aortic smooth muscle cells; Pi, inorganic phosphorous; HPSI, siRNA and high Pi; HPOE, overexpression and high Pi; MTT, 3-(4,5-dimethyl-2-thiazolyl)-2,5-diphenyl-2H-tetrazolium bromide; OD, optical density.

HIF-1*α* siRNA and overexpression plasmid sequences were employed to regulate HIF-1*α* expression; western blotting was used to detect HIF-1*α* protein expression levels in cultured HASMCs for 24 h following transfection (Figure 1). The results indicated that the relative expression levels of HIF-1*α* were significantly inhibited by HIF-1*α* siRNA compared with those in the CNT and scramble siRNA groups (*p* < 0.01) (Figure 1(a)). Moreover, HIF-1*α* expression was significantly upregulated following treatment of the cells with a HIF-1*α* overexpression plasmid compared with that noted in the CNT and empty plasmid groups (*p* < 0.05; Figure 1(b)), indicating the validity of the HIF-1*α* gene regulation. The knockdown efficacy was also detected on day 7. As shown in Figures 2(a) and 2(b), both the expression levels of HIF-1*α* protein and mRNA were significantly inhibited (*p* < 0.01).

### 3.2. Effects of HIF-1*α* Regulation on Cell Growth and Pit-1 Expression in High Phosphate-Stimulated HASMCs

**Figure 3:**
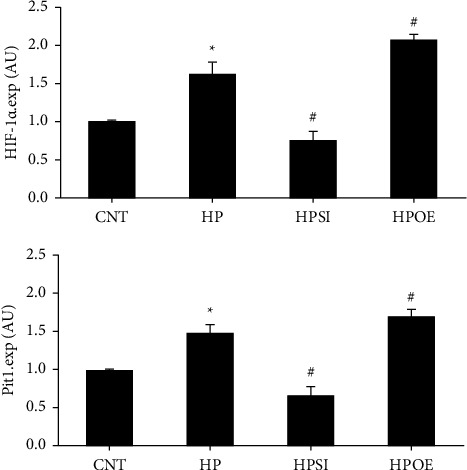
The changes in the gene expression levels of HIF-1*α* and Pit-1. RT-qPCR analysis was employed to examine the relative mRNA expression levels of HIF-1*α* and Pit-1 on day 7. The data shown are indicative of mean ± SD. ^*∗*^*p* < 0.05 vs. CNT; ^**#**^*p* < 0.05 vs. HP. CNT, HASMCs cultured under normal conditions with 0.9 mM Pi; HP, HASMCs cultured with 2.5 mM Pi; HPSI, HIF-1*α* siRNA-transfected HASMCs cultured with 2.5 mM Pi; HPOE, HIF-1*α* overexpression plasmid-transfected HASMCs cultured with 2.5 mM Pi. HIF-1*α*, hypoxia-inducible factor 1 alpha; pit-1, type III sodium-dependent phosphate cotransporter 1; RT-qPCR, reverse transcription-quantitative PCR; SD, standard deviation; CNT, control; HP, high Pi; HASMCs, human aortic smooth muscle cells; Pi, inorganic phosphorous; HPSI, siRNA and high Pi; HPOE, overexpression and high Pi.

**Figure 4:**
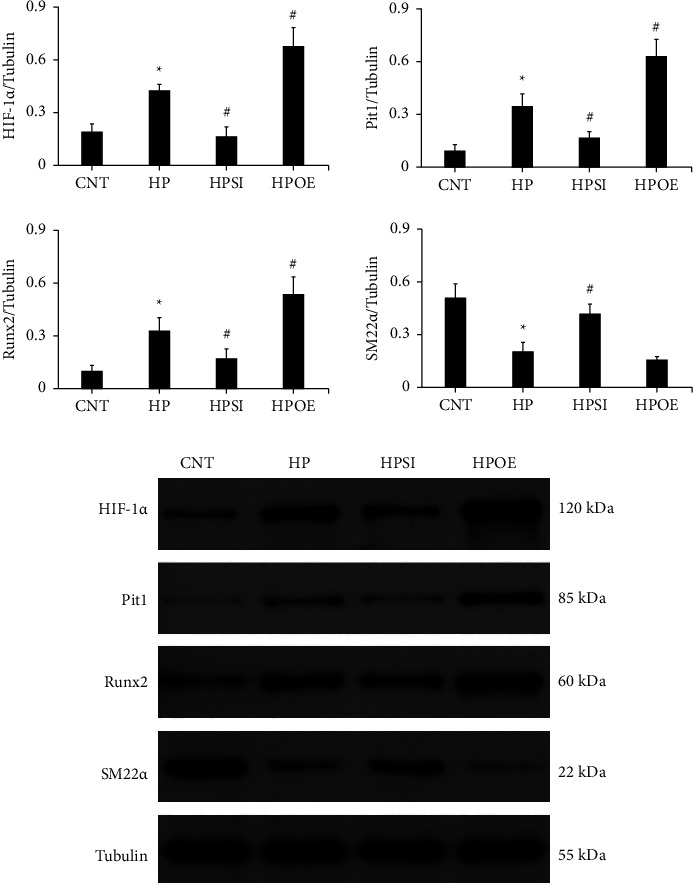
The changes in the protein expression levels of HIF-1*α*, Pit-1, Runx2, and SM22*α*. Western blot analysis was employed to examine the protein expression levels of HIF-1*α*, Pit-1, Runx2, and SM22*α* on day 7. The levels were normalized to those of tubulin. The data shown are indicative of mean ± SD. ^*∗*^*p* < 0.05 vs. CNT; ^#^*p* < 0.05 vs. HP. CNT, HASMCs cultured under normal conditions with 0.9 mM Pi; HP, HASMCs cultured with 2.5 mM Pi; HPSI, HIF-1*α* siRNA-transfected HASMCs cultured with 2.5 mM Pi; HPOE, HIF-1*α* overexpression plasmid-transfected HASMCs cultured with 2.5 mM Pi. HIF-1*α*, hypoxia-inducible factor 1 alpha; Pit-1, type III sodium-dependent phosphate cotransporter 1; Runx2, runt-related transcription factor 2; SM22*α*, smooth muscle 22 alpha; SD, standard deviation; CNT, control; HP, high Pi; HASMCs, human aortic smooth muscle cells; Pi, inorganic phosphorous; HPSI, siRNA and high Pi; HPOE, overexpression and high Pi.

Cell growth was assessed using an MTT assay (Figure 2(c)). The results indicated that the addition of 2.5 mM phosphate or upregulation of HIF-1*α* promoted cell growth (*p* < 0.05), while the knockdown of HIF-1*α* expression inhibited cell growth (*p* < 0.05). The RT-qPCR assay was performed to analyze the mRNA expression levels of HIF-1*α* and Pit-1 on day 7 (Figure 3), and western blotting was used to detect HIF-1*α* and Pit1 protein expression levels (Figure 4). The results indicated that the expression levels of HIF-1*α* and Pit-1 were significantly upregulated in the HP group compared with those in the CNT group (*p* < 0.05). It is interesting to note that Pit-1 expression levels were significantly suppressed in the HPSI group and significantly increased in the HPOE group compared with those in the HP group (*p* < 0.05), which indicated a molecular regulation between HIF-1*α* and Pit-1. Therefore, the regulatory capacity of HIF-1*α* on Pit-1 was demonstrated under high phosphate conditions.

### 3.3. Effects of HIF-1*α* Regulation on the Phenotypic Transition Marker Genes in High Phosphate-Stimulated HASMCs

**Figure 5:**
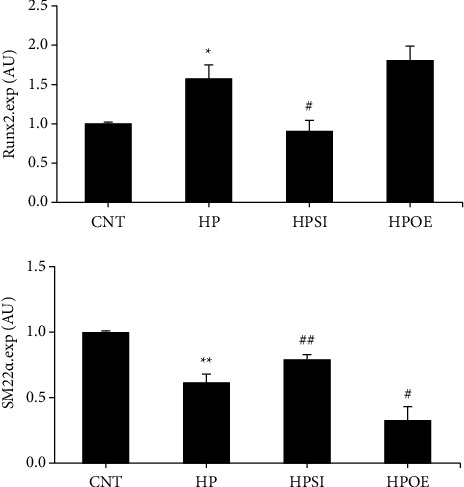
Changes in cell phenotype. The relative mRNA expression levels of Runx2 and SM22*α* on day 7 were detected to trace the phenotypic transition of cultured HASMCs by RT-qPCR analysis. The data shown are indicative of mean ± SD. ^*∗*^*p* < 0.05 vs. CNT; ^*∗∗*^*p* < 0.01 vs. CNT; ^**#**^*p* < 0.05 vs. HP; ^**##**^*p* < 0.01 vs. HP. CNT, HASMCs cultured under normal conditions with 0.9 mM Pi; HP, HASMCs cultured with 2.5 mM Pi; HPSI, HIF-1*α* siRNA-transfected HASMCs cultured with 2.5 mM Pi; HPOE, HIF-1*α* overexpression plasmid-transfected HASMCs cultured with 2.5 mM Pi. Runx2, runt-related transcription factor 2; SM22*α*, smooth muscle 22 alpha; HASMCs, human aortic smooth muscle cells; RT-qPCR, reverse transcription-quantitative PCR; SD, standard deviation; CNT, control; HP, high Pi; Pi, inorganic phosphorous; HPSI, siRNA and high Pi; HIF-1*α*, hypoxia-inducible factor 1 alpha; HPOE, overexpression and high Pi.

In addition to the previous findings, the expression levels of the phenotypic transition marker genes, runt-related transcription factor 2 (Runx2), and smooth muscle 22 alpha (SM22*α*) were evaluated in high phosphate-treated HASMCs on day 7. RT-qPCR analysis (Figure 5) and western blotting (Figure 4) demonstrated the upregulation of Runx2 (*p* < 0.05) and the downregulation of SM22*α*(*p* < 0.01) in the HP group compared to the corresponding levels noted in the CNT group, which indirectly reflected the phenotypic transition of the cultured cells to a certain extent. Knockdown of HIF-1*α* expression resulted in a significant attenuation of the alterations in Runx2 and SM22*α* expression levels induced by high phosphate (*p* < 0.05). In the HPOE group, notable changes in the expression levels of Runx2 (*p*=0.19) and SM22*α*(*p* < 0.05) were noted compared with those of the HP group.

### 3.4. HIF-1*α* Regulates High Phosphate-Induced VC via Pit-1

**Figure 6:**
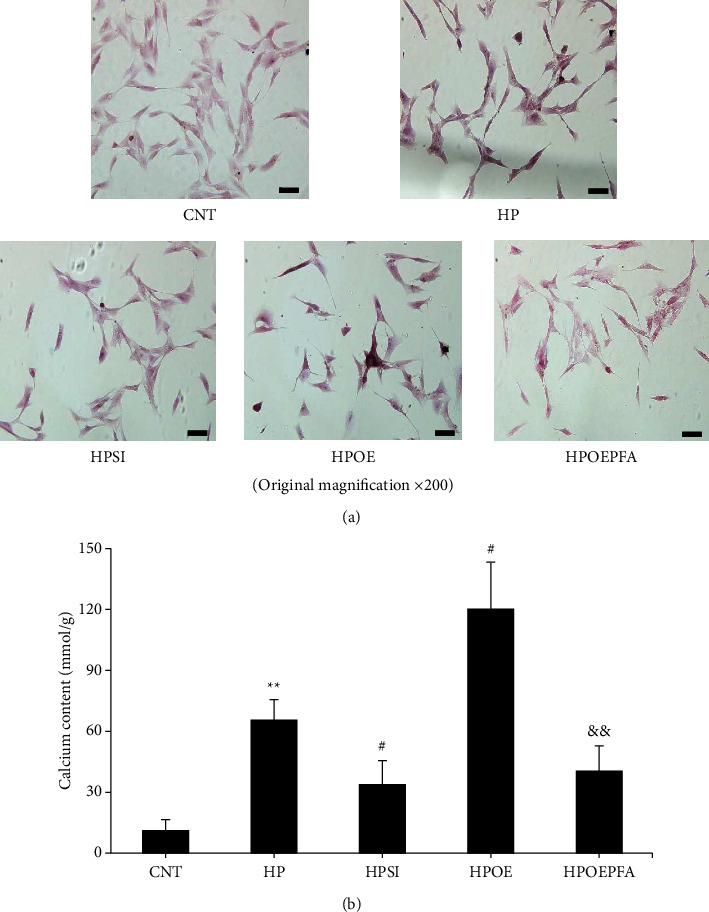
Changes in calcium deposition. (a) Reddish brown coloration by Alizarin red staining indicates calcium deposits in cultured HASMCs (magnification ×200). (b) The quantitative evaluation of calcium deposition in cultured HASMCs was examined by the o-cresolphthalein complexone method. The data shown are indicative of mean ± SD. ^*∗∗*^*p* < 0.01 vs. CNT; ^**#**^*p* < 0.05 vs. HP; ^**&&**^*p* < 0.01 vs. HPOE. CNT, HASMCs cultured under normal conditions with 0.9 mM Pi; HP, HASMCs cultured with 2.5 mM Pi; HPSI, HIF-1*α* siRNA-transfected HASMCs cultured with 2.5 mM Pi; HPOE, HIF-1*α* overexpression plasmid-transfected HASMCs cultured with 2.5 mM Pi; HPOEPFA, HIF-1*α* overexpression plasmid-transfected HASMCs cultured with 2.5 mM Pi and 0.5 mM PFA. HASMCs, human aortic smooth muscle cells; SD, standard deviation; CNT, control; HP, high Pi; HPOE, overexpression and high Pi; Pi, inorganic phosphorous; HPSI, siRNA and high Pi; HIF-1*α*, hypoxia-inducible factor 1 alpha; siRNA, small interfering RNA; HPOEPFA, overexpression high Pi and phosphonoformic acid group; PFA, phosphonoformic acid. Scale bar = 20 *μ*m.

Alizarin red staining (Figure 6(a)) and the o-cresolphthalein complexone method (Figure 6(b)) were employed to determine the calcification degree and investigate the underlying mechanism of cultured HASMCs on day 7. The morphological and quantitative measurements indicated that high phosphate conditions induced significant calcification compared with normal conditions (*p* < 0.01). Moreover, the calcification degree was significantly alleviated in the HPSI group (*p* < 0.05) and aggravated in the HPOE group (*p* < 0.05) compared with that noted in the HP group. Most notably, PFA, a specific antagonist of Pit-1, significantly disrupted the calcification of HASMCs transfected with a HIF-1*α* overexpression plasmid (*p* < 0.01). These results indicate that HIF-1*α* facilitates high phosphate-induced VC via Pit-1.

## 4. Discussion

In the present study, the expression levels of HIF-1*α* and Pit-1 were upregulated in high phosphate-stimulated HASMCs. Upon inhibition of HIF-1*α* expression, a corresponding decrease in Pit-1 expression was observed, and *vice versa*. These findings provide evidence for the regulatory capacity of HIF-1*α* on Pit-1, suggesting that the activation of the HIF-1*α*/Pit-1 signaling pathway may occur upon high phosphate stimulation. Further investigations are required to clarify these findings. To the best of our knowledge, the present study is the first to explore the interaction between HIF-1*α* and Pit-1.

Hyperphosphatemia has been shown to accelerate VC [19, 20]; however, the underlying mechanisms require clarification [4]. Mokas et al. [16] initially reported the pro-calcifying characteristic of HIF-1*α* in high phosphate-induced VC. To date, the mechanistic link between HIF-1*α* and high phosphate-induced VC is still unclear. Pit-1 is the predominant NaPi cotransporter in human VSMCs [21], which has been identified as a pivotal transporter in phosphate-induced VC [11]. Phosphate can upregulate Pit-1 expression and its activity in VSMCs [22]. Previous studies conducted by our group have also supported the significant role of Pit-1 in high phosphate-induced VC [12, 13, 17]. The present findings indicate that HIF-1*α* may fulfill its pro-calcifying characteristic via its regulation on Pit-1. It is concluded that HIF-1*α* as well as Pit-1 may become therapeutic targets for high phosphate-induced VC. In addition, it is inferred that HIF-1 strengthening agents may exert negative effects on the cardiovascular health of patients with CKD and that their clinical use should be cautious in the specific population subgroups. Consequently, high-quality real-world studies should be carried out to evaluate this effect.

To further verify the findings of the present study, the expression levels of the phenotypic transition marker genes, Runx2 and SM22*α*, were investigated in cultured HASMCs. As demonstrated in our previous investigations, the upregulation of Runx2 concomitant with the downregulation of SM22*α* specifically occurs during the phenotypic transition of HASMCs from smooth muscle cells to osteoblast-like cells [12, 13, 17]. The expression of phenotypic transition marker genes in HASMCs cultured under high phosphate conditions was blunted upon transfection with HIF-1*α* siRNA, while it was exacerbated following transfection with a HIF-1*α* overexpression plasmid. These results support the notion that HIF-1*α* can modulate the phenotypic transition of vascular smooth muscle cells, which was reported by other investigations [23, 24]. However, whether HIF-1*α* modulates the phenotypic transition through Pit-1 or other molecules requires additional verification.

The lack of experiments checking the effect of PFA alone on calcification is a limitation of the present study, as it is beyond our scope. Additionally, *Villa-Bellosta R.* and *Sorribas V.* reported that PFA alone prevents high phosphate-induced calcification [21]. Therefore, we believe that PFA alone can restrict calcification in our experiments. The key role of Pit-1 in high phosphate-induced VC has been extensively investigated [10–13, 17, 25]; therefore, we did not assess the impact of Pit-1 overexpression without HIF-1a activation, which may be considered a limitation of this study. Another limitation is that we did not check the mRNA expression of Runx2 and SM22*α* in the HPOEPFA group, which represents a potential avenue for future investigation.

To the best of our knowledge, the present study is the first to confirm that HIF-1*α* regulates Pit-1 expression and exerts its pro-calcifying effect through Pit1. The findings unveil HIF-1*α* and Pit-1 as therapeutic targets for high phosphate-induced VC.

## Data Availability

All data generated and analyzed during the current study are available from the corresponding author upon reasonable request.
